# Supplementary material for: A Novel Frameshift CHD4 Variant Leading to Sifrim-Hitz-Weiss Syndrome in a Proband with a Subclinical Familial t(17;19) and a Large dup(2)(q14.3q21.1)
Source: Biomedicines. 2022 Dec 21;11(1):12. doi: 10.3390/biomedicines11010012 (PMC9855399; doi:10.3390/biomedicines11010012)
Supplement: Supplementary file 1 [file biomedicines-11-00012-s001.zip › Figure S1.pdf]

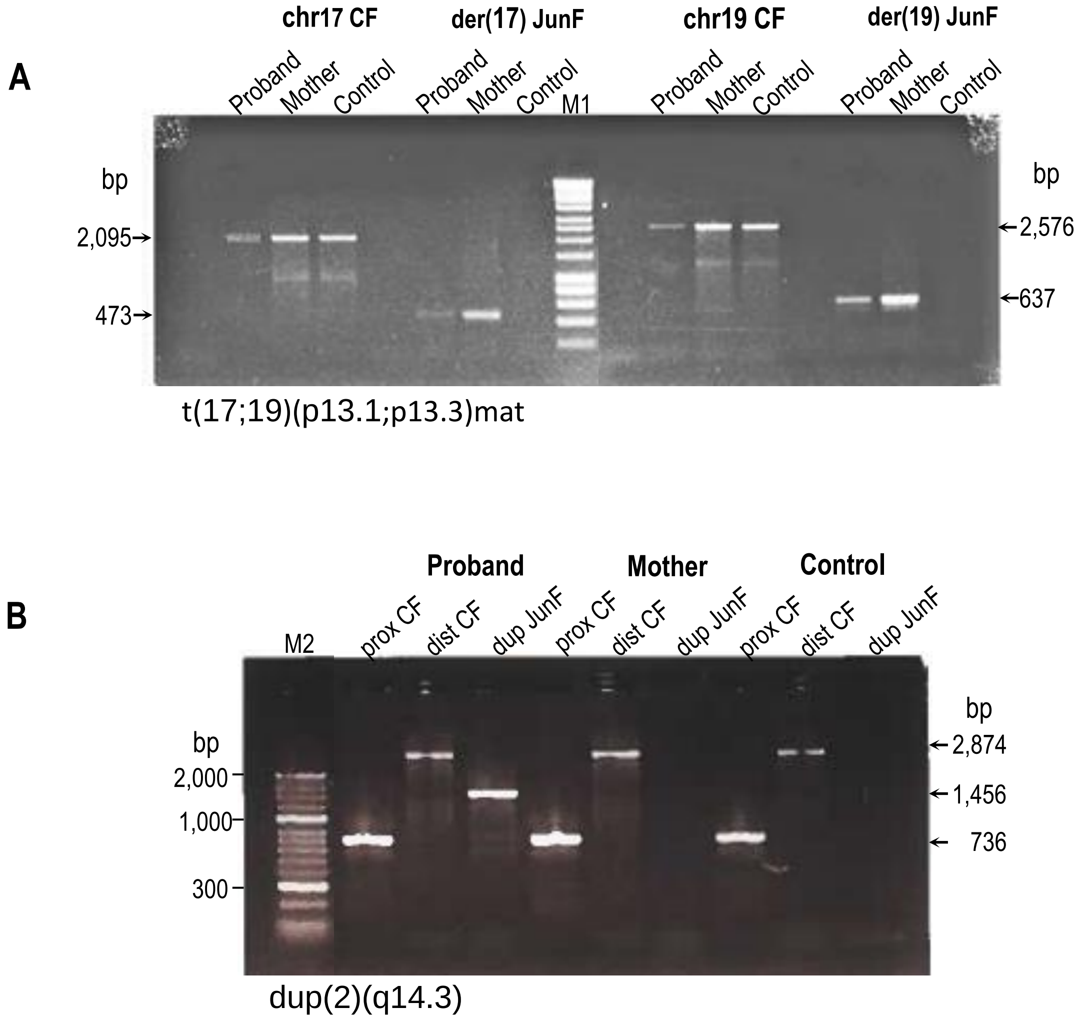

Figure S1. Familial segregation study. **(A)** Segregation analysis of the familial t(17;19)(p13.1;p13.3). Control fragments of 2,095 bp and 2,576 bp and translocation-specific junction fragments of 473 bp and 637 bp were amplified from chr17 , chr19, der(17) and der(19), respectively. chr17 CF – chromosome 17 control fragment; chr19 CF – chromosome 19 control fragment; der(17) JunF – der(17) junction fragment; der(19) JunF – der(19) junction fragment; M1 – size marker HyperLadder I. Control and junction fragments are indicated by arrows.

(B) Segregation analysis of the 3,195,005 bp dup(2)(q14.3). A 736 bp and a 2,874 bp control fragments were amplified from the proximal and distal duplication breakpoint regions, respectively. Whereas, the duplication-specific junction fragment is 1,456 bp in size. prox CF – proximal control fragment and dist CF – distal control fragment; dup JunF – duplication-specific junction fragment; M2 – size marker HyperLadder II.
