# Supplementary material for: A Novel Frameshift CHD4 Variant Leading to Sifrim-Hitz-Weiss Syndrome in a Proband with a Subclinical Familial t(17;19) and a Large dup(2)(q14.3q21.1)
Source: Biomedicines. 2022 Dec 21;11(1):12. doi: 10.3390/biomedicines11010012 (PMC9855399; doi:10.3390/biomedicines11010012)
Supplement: Supplementary file 1 [file biomedicines-11-00012-s001.zip › Table S1.pdf]

Table S1: Primers used for validation of variants breakpoints in case DGRC0021

Fragments	Designation	Primer sequence (5′-3′)	Primer localization	Annealing T (Cº)	Amplicon length (bp)
**t(17;19)(p13.1;p13.3) – Familiar study and SS**					
Control frag. chr17	AC027045-2F	AAGCCATGTGAGCCAAGTTCT	chr17:9,817,830-9,817,850	60	2,095
	AC026591-2R	AAAAAGAATACAGGCCAGGCG	chr17:9,819,904-9,819,924		
Control frag. chr19	AC010503-1F	CTTATAGCCCCACTATGCCCAG	chr19:6,569,707-6,569,728	60	2,576
	AC010503-2R	GGAGTGCTCTGGGTCCCTAA	chr19:6,572,263-6,572,282		
Junction frag. der(17)	AC010503-1F	CTTATAGCCCCACTATGCCCAG	chr19:6,569,707-6,569,728	60	1,719
	AC026591-2R	AAAAAGAATACAGGCCAGGCG	chr17:9,819,904-9,819,924		
Junction frag. der(19)	AC027045-1F	CCTGGCCTTTCCACTGTTTTG	chr17:9,814,227-9,814,247	60	639
	AC010503-4R	GCTCCTGATTTGCCTGGTTTG	chr19:6,573,582-6,573,602		
**dup(2)(q14.3) of 3,195 kb – Familiar study**					
Control frag. chr2 proximal	AC020547-1F	GTCAGACGGTATGTGAGAGCA	chr2:125,920,373-12,5920,393	60	736
	AC020547-4R	CCTTTAGCAGGGTTGTGGACC	chr2:125,921,088-125,921,108		
Control frag. chr2 distal	AC068483-5F	TCCAGTGGGAACTACCCATCTAT	chr2:129,114,655-129,114,677	60	2,874
	AC068483-6R	CTGAACATGCCAAGCATCCG	chr2:129,117,509-129,117,528		
Junction frag. dup(2)	AC068483-5F	TCCAGTGGGAACTACCCATCTAT	chr2:129,114,655-129,114,677	60	1,456
	AC020547-4R	CCTTTAGCAGGGTTGTGGACC	chr2:125,921,088-125,921,108		
**dup(2)(q14.3) of 3,195 kb – SS**					
Control frag. chr2 proximal	AC020547-1F	GTCAGACGGTATGTGAGAGCA	chr2:125,920,373-12,5920,393	60	464
	SS_AC020547-6R	CTGCCTCAGCCATTCTACGG	chr2:125,920,815-125,920,834		
Control frag. chr2 distal	SS_AC068483-8F	GATCGAGAATCCTGGAGCCAC	chr2:129,115,309-129,115,329	60	2,220
	AC068483-6R	CTGAACATGCCAAGCATCCG	chr2:129,117,509-129,117,528		
Junction frag. dup(2)	SS_AC068483-8F	GATCGAGAATCCTGGAGCCAC	chr2:129,115,309-129,115,329	60	530
	SS_AC020547-6R	CTGCCTCAGCCATTCTACGG	chr2:125,920,815-125,920,834		

[a]Reference human genome assembly is GRCh38/hg38; SS - Sanger sequencing; Frag. – Fragment. Primers with the prefix SS were specifically used for SS.
