# Supplementary material for: A Novel Frameshift CHD4 Variant Leading to Sifrim-Hitz-Weiss Syndrome in a Proband with a Subclinical Familial t(17;19) and a Large dup(2)(q14.3q21.1)
Source: Biomedicines. 2022 Dec 21;11(1):12. doi: 10.3390/biomedicines11010012 (PMC9855399; doi:10.3390/biomedicines11010012)
Supplement: Supplementary file 1 [file biomedicines-11-00012-s001.zip › Table S6.pdf]

Table S6: Comparison of the proband's (DGRC0021) clinical features based on the phenotype similarity score

Proband vs	PhenSSc	P	MaxSSc	MaxDiseaseSSc
OMIM #617159	1.29	0.1960	3.02	3.64
All variants (n=44)	2.33	0.0587	3.02	4.20
Truncating variants (n=4)	1.21	0.0653	3.02	3.32
Missense variants (n=38)	1.22	0.0100	3.02	1.97
Splicing variants (n=2)	0.81	0.1550	3.02	5.24

Comparisons are made against Sifrim-Hitz-Weiss syndrome clinical synopsis at OMIM #617159, and four categories of pathogenic variants according to Table S1. PhenSSc – Phenotype similarity score; MaxSSc –Maximum similarity score; MaxDiseaseSSc – Maximum disease similarity score.
